# Interspecific trait variability and local soil conditions modulate grassland model community responses to climate

**DOI:** 10.1002/ece3.8513

**Published:** 2022-02-22

**Authors:** Franklin Alongi, Jana H. Rüthers, Justyna Giejsztowt, Katrina LaPaglia, Anke Jentsch

**Affiliations:** ^1^ Department of Disturbance Ecology BayCEER University of Bayreuth Bayreuth Germany; ^2^ Department of Plant Science and Plant Pathology Montana State University Bozeman Montana USA

**Keywords:** climate change, community ecology, grassland ecology, mountain ecosystems, species asynchrony

## Abstract

Medium‐to‐high elevation grasslands provide critical services in agriculture and ecosystem stabilization, through high biodiversity and providing food for wildlife. However, these ecosystems face elevated risks of disruption due to predicted soil and climate changes. Separating the effects of soil and climate, however, is difficult *in situ*, with previous experiments focusing largely on monocultures instead of natural grassland communities. We experimentally exposed model grassland communities, comprised of three species grown on either local or reference soil, to varied climatic environments along an elevational gradient in the European Alps, measuring the effects on species and community traits. Although species‐specific biomass varied across soil and climate, species' proportional contributions to community‐level biomass production remained consistent. Where species experienced low survivorship, species‐level biomass production was maintained through increased productivity of surviving individuals; however, maximum species‐level biomass was obtained under high survivorship. Species responded directionally to climatic variation, spatially separating differentially by plant traits (including height, reproduction, biomass, survival, leaf dry weight, and leaf area) consistently across all climates. Local soil variation drove stochastic trait responses across all species, with high levels of interactions occurring between site and species. This soil variability obscured climate‐driven responses: we recorded no directional trait responses for soil‐corrected traits like observed for climate‐corrected traits. Our species‐based approach contributes to our understanding of grassland community stabilization and suggests that these communities show some stability under climatic variation.

## Graphical abstract

Climate and soil effects are often inseparable, and are expected to become highly variable under future climate models. In our study, we observe plant model grassland communities throughout the European Alps to determine the effects of climate and soil variation on species trait dynamics, revealing different responses.

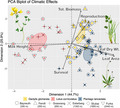

## 1 INTRODUCTION

Grasslands cover a quarter of Earth's terrestrial surface, having both agricultural and conservational importance (White et al., 2000). Grassland communities sequester carbon, protect soil against erosion, and supply nutrient‐rich feed for agriculture (Zhao et al., 2020). High‐elevation grasslands are particularly known for being both highly endemic and having high species richness at regional scales, making them communities of high interest for biodiversity conservation (Gillet et al., 2016; Körner, 2003, 2004). These alpine regions, however, are at an especially high risk of disturbance from climate change (Schirpke et al., 2017). European alpine temperatures are expected to increase at above‐average rates due to climate change (Gobiet et al., 2014; Li et al., 2019; Mountain Research Initiative EDW Working Group, 2015). Surface air temperature in the European Alps is rising at 0.3 ± 0.2 °C per decade, exceeding global warming trends (Hock et al., 2019). Rising temperatures have implications for plant functional traits (hereafter “trait”), such as biomass, leaf area, and reproduction which have direct implications for overall plant fitness (Alexander et al., 2015; Debouk et al., 2015; Wipf et al., 2006). Such warming can lead to community instability by increasing species synchrony (more synchronic responses of species composing the community) (Ma, Liu, et al., 2017). No consensus exists regarding generalized plant community responses to climate change due to complex interactions between climate and soil compositions (Yang et al., 2018). Measures such as trait responses offer an improved understanding of ecological responses that are comparable across regions and experimental approaches (Sporbert et al., 2021; Vandvik et al., 2020).

Medium‐to‐high elevation grassland communities are vulnerable to climate change, in part, because these specialists perform poorly when faced with increased competition from invading lowland species (Alexander et al., 2015; Giejsztowt et al., 2020; Hansen et al., 2021; Smithers et al., 2021). Community responses can vary because temperature affects both competitive and facilitative processes within semi‐natural grassland ecosystems (Olsen et al., 2016). While some studies have correlated rising temperature to increases in aboveground community biomass (Berauer et al., 2019; Halbritter et al., 2018; Niu et al., 2019), others have identified no such trend (Fu et al., 2013; Liu et al., 2018), demonstrating the sensitive nature of biomass and other trait responses to climatic variation. While community biomass is a coarse way to compare productivity across communities, a more nuanced understanding of community dynamics is enabled by investigating species‐specific or functional group responses. Elevated temperatures can lower community biomass stability if composing species have asynchronous responses (Ma, Yan, et al., 2017). Dominating species stability has also been identified as a stronger driver of biomass production stability than species richness (Valencia et al., 2020). The stability of biomass production has immediate consequences for human activities such as agriculture as well as implications for long‐term ecosystem function and resistance to stressors like drought (Muraina et al., 2021). Consequently, examining biomass and other intra‐specific trait responses is critical to understanding climate change effects on community‐level productivity.

Although responses to soil characteristics can be species specific, studies measuring community responses to soil variation are nonetheless able to draw general trends (Zas & Alonso, 2002). For example, nutrient addition can destabilize grassland primary production (Bharath et al., 2020). While this could be explained by asynchronous species responses to fertilization, unfortunately, species' trait differences are often omitted from community‐level studies investigating soil effects, which could reveal differential species responses within a community. In contrast, the effects of climate change on both above‐ and belowground physiological traits (above ground biomass, below ground biomass, leaf area, etc.) are well documented, with effects typically mediated by changes in soil composition, fauna, and the microbial community (Briones et al., 2009; Hagedorn et al., 2019). Traits changes can in turn affect soil microbiota, resulting in interdependency of species within a community (Wang et al., 2017). Puissant et al. (2017) projected that climate warming would lead to reduced soil organic carbon content, thus decreasing soil microbial activity, and ultimately lowering plant biomass, while Chen et al. (2020) predicted increases in soil organic carbon as a result of warming. These contrasting findings highlight the dependence of community responses on climate and local soil. Field experiments that manipulate climate while incorporating natural soil variation will therefore more accurately predict trait responses in plant grassland communities than observational studies that cannot partition the effects of these drivers.

Plant communities will experience changes in several abiotic parameters due to climate change, such as precipitation, seasonality, and temperature regimes, resulting in altered biotic conditions. For species to cope with climatic changes, interspecific trait variation, phenotypic plasticity, and local adaptations are essential (Frei et al., 2014; Gonzalo‐Turpin & Hazard, 2009; Midolo & Wellstein, 2020). Grassland species generally respond plastically to changing environmental conditions (Cui et al., 2018; Kreyling et al., 2019; Valladares et al., 2014), however, co‐occurring grassland species exhibit differences in trait responses to climatic stress (Hamdani et al., 2019). While it can be expected that species respond differently under stress, how these species‐dependent responses affect overall community trends remains unclear.

Here, we monitored model grassland communities in the European Alps for one year. By experimentally manipulating both soil composition and climate, we identified the independent effects of each driver on species‐ and community‐level traits. We measured a variety of traits related to productivity and fitness. Specifically, we hypothesized that (1) the relative contribution of individuals and species to total community biomass would remain constant irrespective of community productivity, (2) climate and soil differences would lead to trait variation across species and locations, and (3) our community‐based approach would identify separable effects of climate and soil on plant trait dynamics.

## 2 MATERIALS AND METHODS

### 2.1 Experimental design

Our experiment investigated community responses to soil and climate variation using standardized communities composed of three species: *Dactylis glomerata* L. (graminoid), *Plantago lanceolata* L. (non‐leguminous forb), and *Lotus corniculatus* L (nitrogen‐fixing legume). Species were selected based on wide climatic tolerances and a global distribution, being naturalized on six continents (Seipel et al., 2012). These species are considered non‐invasive, making them attractive for coordinated studies (Alexander & Edwards, 2010). The experimental communities represent a variety of herbaceous life‐forms, with limited functional overlap (Díaz & Cabido, 2001). These species are well suited to experimental studies due to relatively short life cycles and being readily manipulated. Consequently, these species can be used as a common currency for plant community dynamics across coordinated studies.

**FIGURE 1:**
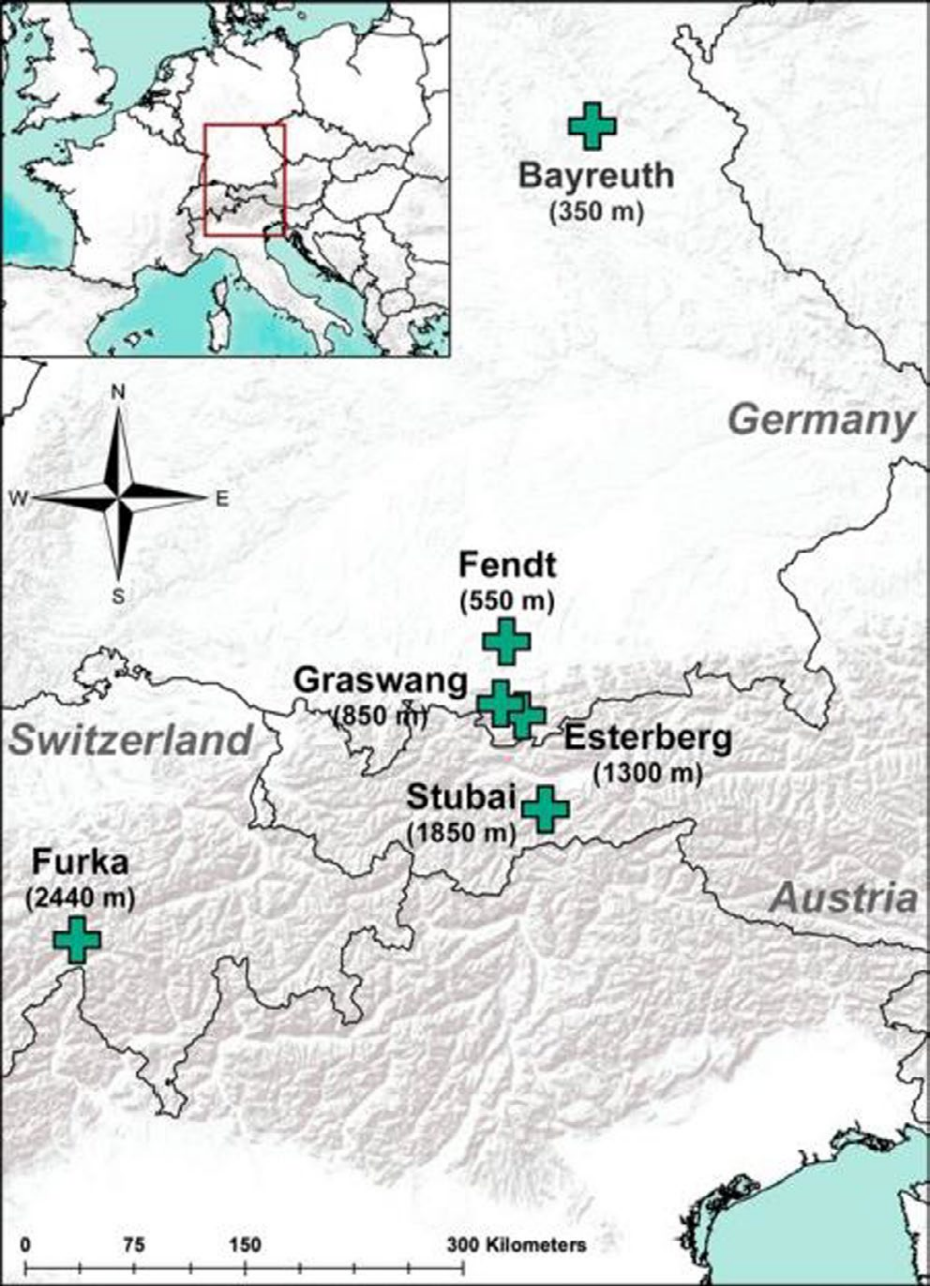
Location of experimental climates across Germany, Austria, and Switzerland. Elevations are reported as meters above sea level

Seeds used in this study were sourced from Rieger‐Hofmann in central Germany to reduce variability in genetic origin. We partitioned the effects of local soil from climatic effects by including a reference substrate treatment (vermiculite mixed with 4g Osmocote fertilizer) at each site. Vermiculite is a suitable substrate comparable to potting soil (Wilfahrt et al., 2021). Plants used in the experiment were reared in a greenhouse in Bayreuth, Germany for 4 weeks before being transported to field sites in summer 2017 (Figure 1). The selected locations were Bayreuth, Germany (350 m a.s.l.); Fendt, Germany (550 m a.s.l.); Graswang, Germany (850 m a.s.l.); Esterberg, Germany (1,300 m a.s.l.); Stubai, Austria (1,850 m a.s.l.); and Furka, Switzerland (2,440 m a.s.l.), and range from medium to high elevation. These locations represent a wide geographical, climatic, and soil compositional range (Table 1, soil composition values from Ingrisch et al., 2018; Steinwandter et al., 2017). High variation in local soil composition represents varying levels of nutrient and water availability. For example, Bayreuth would be expected to have the highest drainage and lowest nutrient retention due to relatively high sand composition, whereas Graswang would be expected to have the lowest drainage and high nutrient retention due to low sand and high clay composition.

**TABLE 1:** Range of environmental variables across selected study sites

Site	Elevation (m.a.s.l.)	Total precipitation (mm)	Mean temperature (ºC)	Soil pH	Clay %	Silt %	Sand %
Bayreuth	350	707.36	9.13	5.21	10.40	19.10	67.20
Fendt	550	1,099.76	8.28	5.15	37.25	36.95	25.75
Graswang	850	1,607.10	6.55	6.76	59.70	47.90	2.75
Esterberg	1,300	1,326.40	5.74	6.15	51.25	43.00	5.80
Stubai	1,850	1,329.64	6.10	5.08	13.30	36.20	50.20
Furka	2,440	1,149.40	0.13	4.19	~10	~30	~60
Reference Soil		*Vermiculite mixed with 4g Osmocote fertilizer was used as reference at all sites*					

Note

Precipitation and temperature values represent calendar year 2017 and were collected from local weather stations established at sites. Soil composition values were obtained from published literature for sites other than Furka, which were collected from finger probes of soil samples.

**FIGURE 2:**
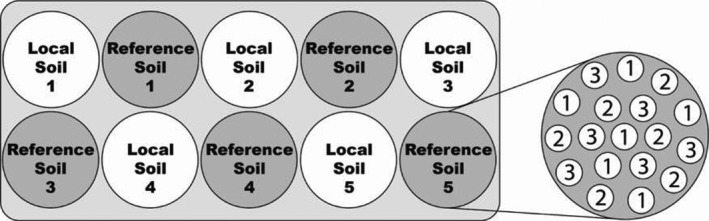
Experimental set‐up of plant communities at each site. Soil type (local or reference) for each community is denoted on the left panel together with replicate number. Each community contained six individuals of each species that were planted pattern‐wise as indicated on the right (1 – *D. glomerata*, 2 – *P. lanceolata*, and 3 – *L. corniculatus*). Note – diagram not drawn to scale and is not intended for exact spatial interpretation

Individuals were transplanted into 11‐liter pots (30 cm diameter × 24 cm depth) containing either the local soil or the reference soil. Six individuals of each species were planted per pot (hereafter: “community”), with five replicates of each soil type per site (Figure 2). Consequently, 1,080 individuals were used in the experiment. Communities were buried into the ground, watered for 10 days, and then left to grow under natural conditions. After 1 year, the counts of surviving (those that survive to the end of experiment) and reproducing individuals (those that produce reproductive structures), as well as the maximum growth height (cm) of each species, were recorded. Five leaves were collected per plant for leaf area (cm^2^) and dry weight (g) measurements. Then, surviving plants were harvested at 3 cm above the soil. Species‐specific aboveground biomass (g) (same species in single community) was weighed and dried at 60°C for 48 h. Community root biomass (g) was harvested, dried, and weighed, as roots of individual species were indiscernible. These traits were selected due to their ease of measurement, common use among ecological experiments, as well as their direct relationship to plant and community fitness (specifically productivity and reproduction).

### 2.2 Statistical methods

All data analysis was both performed and visualized in the R programming environment (version 4.1.1, R Core Team, 2021). Diagnostic plots were used to verify all parametric modeling assumptions, with log transformations being performed when necessary to satisfy assumptions. Linear models were fit using numerical plant traits as response variables (biomass, individual biomass, maximum growth height, leaf area, leaf dry weight, reproduction, and survivorship), with treatments (site, species, and soil type) as predictor variables. Sites were analyzed categorically representing a wide variety of environmental variables, rather than across specific variables. Full models with all interaction combinations, as well as all simplified model structures were compared in the package MuMIn (version 1.43.17, Bartoń, 2020). The best performing model for each response variable was selected based on Akaike's information criterion (AIC; Bozdogan, 1987; Wagenmakers & Farrell, 2004), except where multiple models were indistinguishable (δAIC < 2), in which case the simplest model structure was selected.

To identify which traits were most associated with individual predictors (species, soil, and climate), we created confusion and importance matrices using the package randomForest (version 4.6‐14, Liaw & Wiener, 2001). Out‐of‐bag error rates (OOB) were derived from confusion matrices to estimate the relative error of traits in treatment differentiation. OOB values were standardized through the calculation of percent difference from random classification and are referred to as error decrease. For the three most deterministic variables, mean decrease accuracies corrected by the sample size are reported as percentages, representing the estimate of misclassification that would occur if a variable was removed from the model. Generalized linear models and analysis of variance (ANOVA) models were fit on both top multiple linear regression models as well as physiological variables with high species differentiation power. Tukey's Honest Significant Distance was used post hoc to identify differences across treatments using the package multcomp (version 1.4‐14, Hothorn et al., 2020). This procedure was also used to test biomass differences across climate, species, and soil.

To investigate intra‐specific trends, individual biomass was calculated by dividing species biomasses by the number of survivors of each species. The effects of local soil at a specific climate were isolated by calculating the difference in trait responses between the local and reference soil communities, referred to as soil‐corrected trait values. The effects of climate on plant traits were isolated by comparing reference soil communities across the climates, referred to as climate‐corrected trait values. We used a principal component analysis (PCA) using the package FactoMineR to analyze multivariate data and identify physiological variable contributions to climate and species differences (version 2.4, Lê et al., 2008). Individuals were equally weighted, with variables being positively shifted and logit transformed to standardize relative contributions. PCA dimensional analysis was performed by calculating correlations across maximum height, survival, species biomass, reproduction, leaf dry weight, and leaf area. No derived variables were included in the PCA or dredge modeling to eliminate issues of covariance.

## 3 RESULTS

### 3.1 Biomass production

**FIGURE 3:**
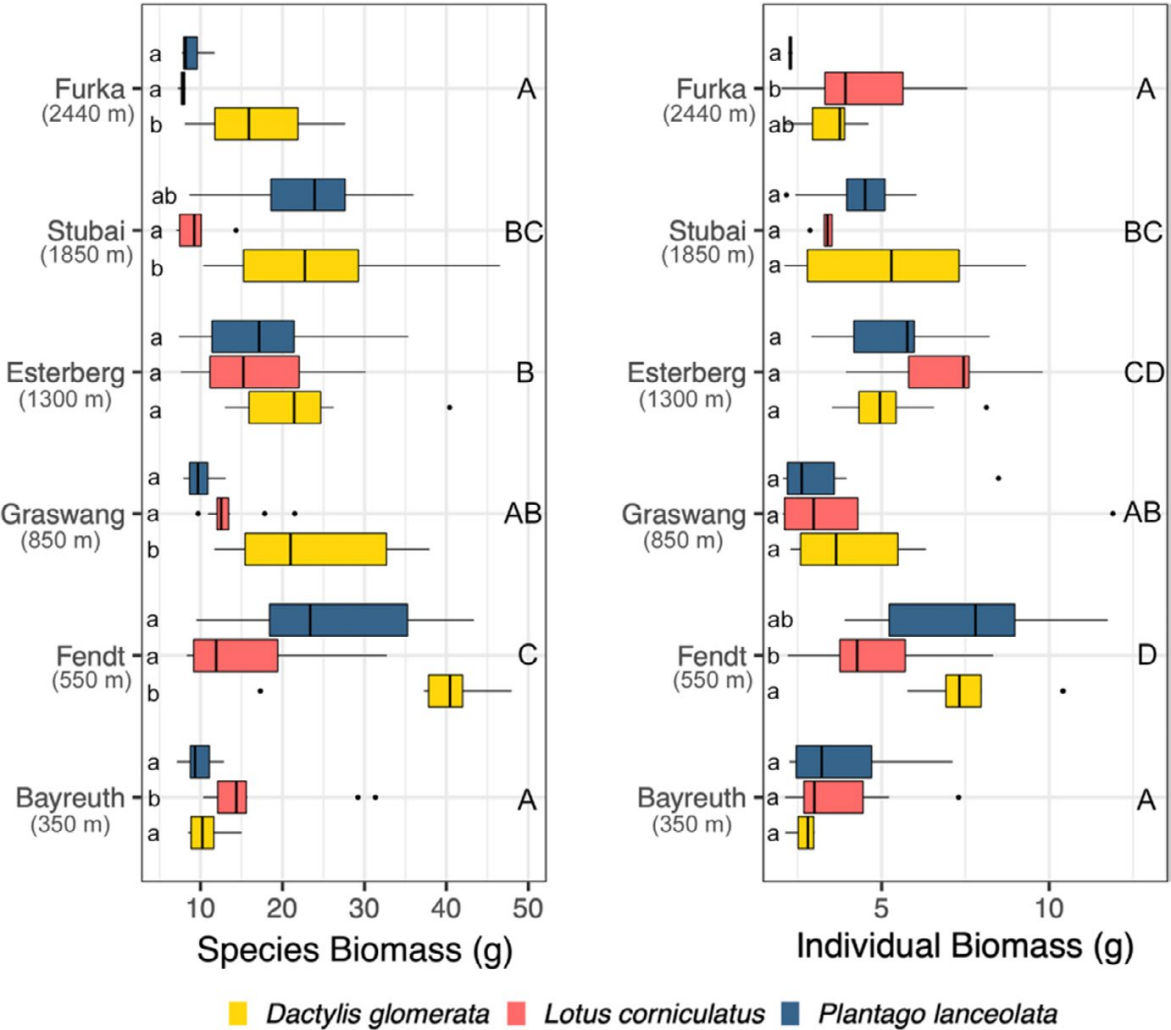
Boxplots representing *Dactylis glomerata* (yellow), *Lotus corniculatus* (red), and *Plantago lanceolata* (blue) biomass response to climate at the species level (left) and individual level (right). Each box is comprised of all 10 communities at that climate and depict biomass interquartile ranges and medians. Lower case letters indicate significant species‐specific differences, while uppercase indicate significant differences between sites

Species biomass was best explained by the full model (δAIC = 7.31) including site, species, an interaction between site and species, an interaction between climate and soil, and a three‐way interaction among climate, species, and soil (*F*_35,133_ = 11.22, *p* < .001, adj‐*R* ^2^ = .680). Prevalence of interactions across all explanatory factors reveals highly contingent biomass dynamics. Lower species‐specific biomass was observed at Bayreuth (350 m) than at Fendt (550 m) and Stubai (1,850 m) (TukeyHSD, *p* < .001, *p* = .016, respectively). All species produced more biomass at Fendt (550 m) compared to Graswang (850 m), Esterberg (1,300 m), Furka (2,440 m) (TukeyHSD, all *p* < .001), and Stubai (1,850 m) (TukeyHSD, *p* = .021). Lower species‐specific biomass occurred at Furka (2,440 m) than at Stubai (1,850 m) (TukeyHSD, *p* = .006). Species differed in biomass production across all climates except Esterberg (1,300 m), with no species consistently producing the most biomass across all sites (Figure 3).

**FIGURE 4:**
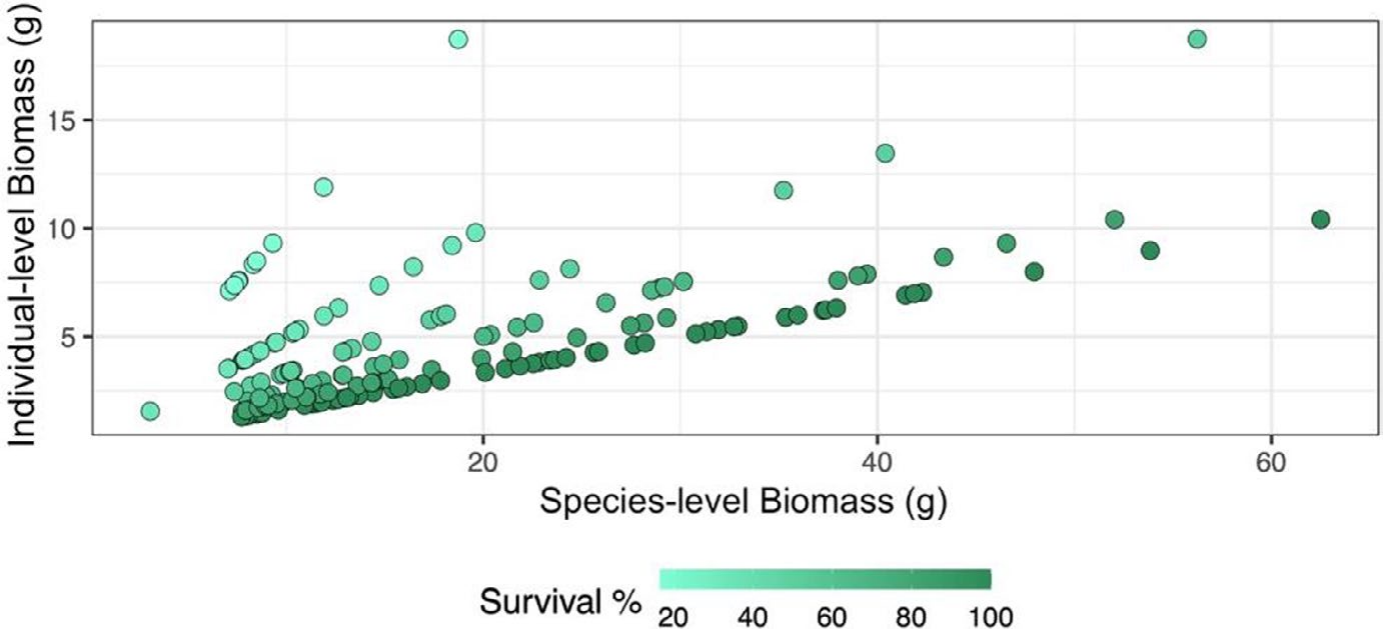
Relationship between individual biomass and species biomass. Each point represents a species within one community. The color gradient represents the survival of that species within the community with dark being high survival. Species groups with the same survival form perfect lines because individual biomass is dependent on species‐level biomass and the species‐specific survival rate within that community

Individual biomass was best explained by the model including climate, soil, an interaction between climate and species, as well as an interaction between soil and species (*F*_20,148_ = 11.22, *p* < .001, adj‐*R* ^2^ = .469). This contrasts with the best performing model explaining community biomass, which was the full model. Interestingly, community biomass hierarchies were generally conserved when measured using individual‐level biomass (unlike at the species level). Individual biomass production differed by species. Different dominance hierarchies were observed when the number of individuals was accounted for than when biomass was pooled at the species level in all sites but Esterberg (1,300 m). While species‐level biomass was not affected by soil type (*F*_1, 167_ = .34, *p* = .562, adj‐*R* ^2^ = −.004), individual biomass was, with reference soil having a higher individual biomass across all climates than local soil (*F*_1, 167_ = 14.85, *p* < .001, adj‐*R* ^2^ = .076). Interestingly, the higher individual biomass corresponded to the reference soil also having a higher root biomass and lower survival when compared to the local soil (*F*_1, 58_ = 7.698, *p* = .007; *F*_1, 58_ = 15.68, *p* < .001, respectively). This revealed an interesting survival‐dependent positive relationship between the individual biomass and the species biomass in each community (Figure 4).

### 3.2 Plant trait trends across soil type, species, and climate

Analyses using *randomForest* were performed using species, community, and site‐level traits, with results being summarized in Table 2. For the species level, analyses were performed with soil type, species identity, and climate as response variables. Overall, species' trait responses were more consistent across climate than soil, with the highest variable predictive ability being observed in association with species. For the community level, analyses were performed with soil and climate as response variables. Once again, climate was observed to have more predictable trait responses than soil. For the site level, analyses were performed with soil type as a response variable.

**TABLE 2:** Summarized outputs of *randomForest* modeling for the species, community, and site‐level variables

Data level	Cat. response	Error decrease	Max. height	Leaf area	Leaf dry Wt.	Survival	Reproducing #	Biomass	Root biomass
Species	Species	38.76%	22.30%	17.80%	16.47%				
	Soil Type	35.93%			4.50%	8.50%	4.00%		
	Climate	38.33%	12.92%	12.10%				16.29%	
Community	Soil Type	26.67%				24.56%	19.72%		20.00%
	Climate	46.63%		39.78%	28.57%	27.68%			
Site	Soil Type	41.63%	18.75%			39.42%	39.17%		

Note

Values for the top three traits are reported for each model run. Plant traits with a percent value represent the estimate of misclassification that would occur if that variable were removed.

### 3.3 Soil effects

**FIGURE 5:**
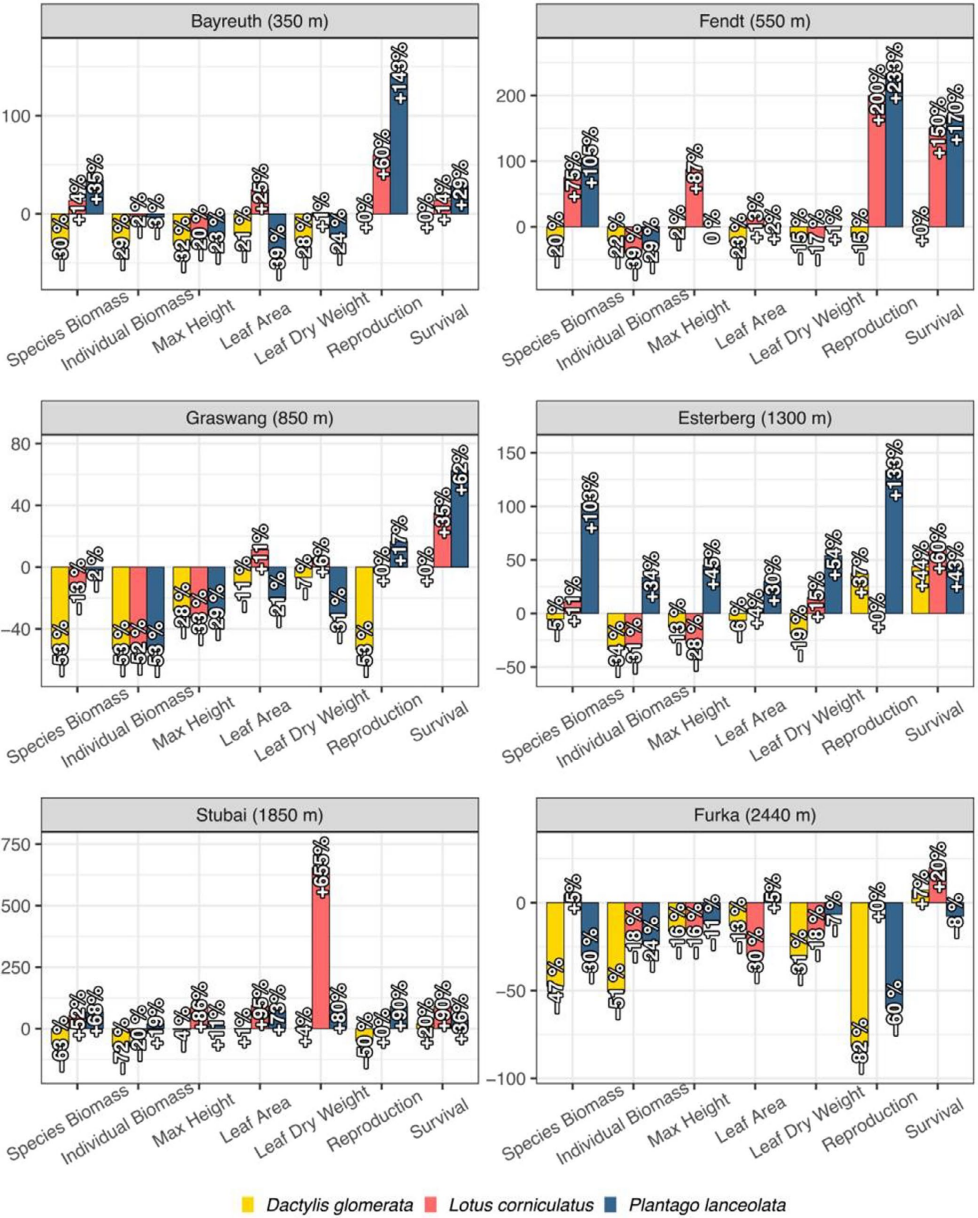
Effects of the local soil at each climate for *D. glomerata* (yellow), *L. corniculatus* (red), and *P. lanceolata* (blue) across several response variables. Y‐axis represents percent change in local soil communities from standard soil communities. Effects of local soil are calculated by subtracting the average response across communities grown on local soil from the average response in communities grown on the reference soil at a given site

Climate‐corrected trait values were used to determine the effect of local soil at each study climate. Explanatory variables at the individual species level included both species and climate, and predictive trait models were characterized by high amounts of interactions, revealing no clear trends across specific climate or species (Figure 5). Species‐specific biomass, individual biomass, maximum height, survival, leaf area, and leaf dry weight were all explained by models containing species, climate, and an interaction between species and climate (Table 3). Only reproduction was explained by models not including an interaction term. Surprisingly, community‐level climate‐corrected analysis revealed no differences in any of the measured physiological variables across any climate (all *p* > .05), indicating that communities responded consistently as a whole regardless of site.

**TABLE 3:** Summarized outputs of linear models across all measured trait response variables

Trait	Optimal model structure	*F* statistic	df	*p* value	δAIC	Adjusted *R* ^2^
Species biomass	Site × Species	13.10	17,69	<.001	28.31	.705
Individual biomass	Site × Species	12.36	17,69	<.001	64.97	.692
Max height	Site × Species	7.31	17,71	<.001	44.78	.633
Survival	Site × Species	3.64	17,72	<.001	4.35	.336
Leaf area	Site × Species	4.93	17,70	<.001	14.50	.434
Leaf dry weight	Site × Species	4.40	17,70	<.001	10.94	.399
Reproduction	Site + Species	13.14	7,82	<.001	3.89	.489

Note

Interpretation: × denotes the presence of the interaction term in the top model, whereas δAIC values represent AIC differences between the additive and interactive model.

### 3.4 Climatic effects

**FIGURE 6:**
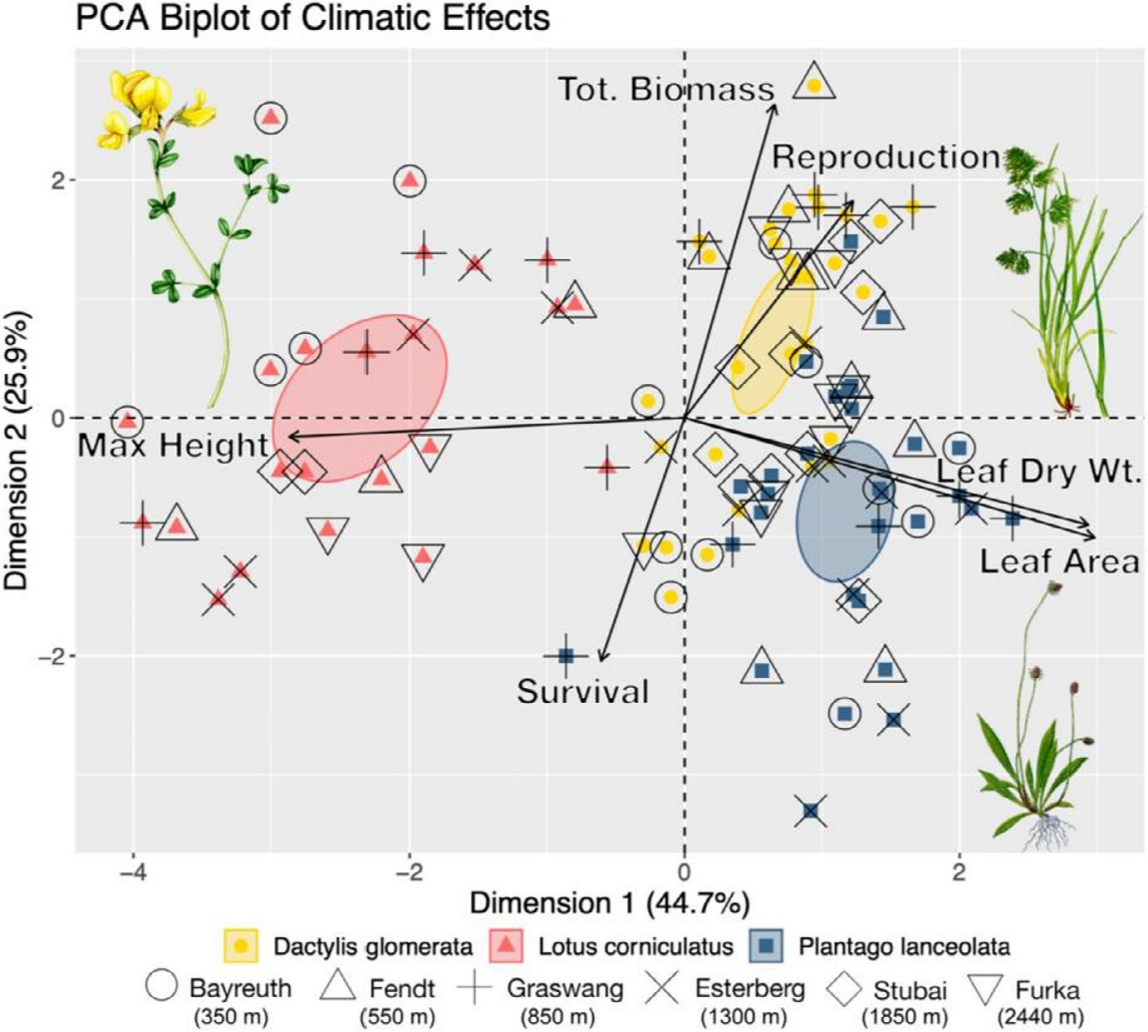
Principal component analysis of the climatic effects on *D. glomerata* (yellow), *L. corniculatus* (red), and *P. lanceolata* (blue) across the six locations. Individuals in reference soil pots were used: the effect of local soil is not displayed. Shaded area represents a 99% confidence interval ellipsoid for each species. Relative effect size of the variable is described by the length of the arrows. Note: Text has been slightly displaced to limit overlap and does not reflect exact ordinal positioning. Illustration of *L. corniculatus* by Lizzie Harper www.lizzieharper.co.uk

The first two dimensions of the PCA resolved 70.6% of the total variance in traits, with the X axis explaining 44.7% and the Y axis explaining 25.9% (Figure 6). The X axis represents the maximum height to leaf dry weight and leaf area traits, while the Y axis represents total biomass (aboveground) and reproduction to survival. Interestingly, traits did not clearly share high correlation across dimensions. Species biomass and reproduction were positively correlated and were both negatively correlated with survival. Leaf dry weight and leaf area were positively and were both negatively correlated with max height. PCA clustering revealed clear differences in trait response between species. *Lotus corniculatus* segregated largely to the negative direction of the first dimension, whereas *D. glomerata* and *P. lanceolata* segregated largely in positive and negative second dimension, respectively. In contrast, climate was not clustered following a PCA analysis – with all climate‐level confidence interval ellipsoids overlapping even at the 5% level.

## 4 DISCUSSION

With this study we aimed to identify the independent effects of soil and climate on plant trait variation within model grassland communities. Our results point to three key findings. The first is that while individual biomass responses to climate and soil were found to be species specific, species‐level dominance hierarchies (i.e., relative species contributions to community biomass) remained stable. Secondly, soil‐corrected trait values revealed that soil differences drove stochastic trait variation across both site and species. Lastly, climate differences lead to relatively consistent trait responses for the three study species, with species separating distinctly in ordinal space across all sites.

While these results exemplify the utility of plant‐model communities as an ecological tool, caution is necessary when interpreting results. Firstly, we lacked statistical power to explore interactions between specific climate and soil effects. We therefore limit interpretations to descriptions of climate‐specific differences and do not treat climates as representations of any single abiotic gradient. To address separating climate effects from soil effects *in situ*, we standardized the local soil to the reference soil at each climate, however, these results must be interpreted in the context of the local climate. Furthermore, our experimental set‐up did not preclude microorganism contamination of the reference soil and we did not characterize the microbial composition of local soils, which possibly affected *L. corniculatus* differentially due to its nitrogen‐fixing abilities. Local soils likely contained rich microbial communities including arbuscular mycorrhizal fungi (AMF). AMF are known symbionts of the three species in this study and affect interspecific competition and growth (Kyriazopoulos et al., 2014; Scheublin et al., 2007). Lastly, it was observed that reference soil treatments had larger root biomass, while concurrently having lower rates of survival, potentially due to physical restriction belowground. With larger root biomass, reference soil communities likely experienced faster dry‐down following precipitation, leaving individuals more susceptible to drought stress (Turner, 2019). This also is a strong indicator of increased belowground competition in reference soil communities, potentially also explaining the lower survival rates in conjunction with drought stress. While these points of concern are common in ecological studies, they nonetheless should be considered when interpreting experimental results.

### 4.1 Overall biomass

Biomass is the most common and coarsest measure of community productivity in grassland ecosystems and is of immediate interest for agriculture. While we found differences in community biomass across our climates characterized by different soils, the species‐specific contributions to community biomass within these climates were consistent: as expected, *D. glomerata*, the graminoid species, typically produced the most biomass with *P. lanceolata* and *L. corniculatus* being less productive. Species‐specific biomass scaled with the community biomass across climates. While this may suggest that species do have consistent contributions to community biomass, comparing survivorship‐corrected (individual) biomasses across species and climates offers a different perspective.

Individual biomass analysis revealed a similar overall relationship as species biomass between climates, showing community hierarchies were largely maintained when accounting for survivorship. However, when looking at individual biomass, different intra‐climate hierarchies emerge than when looking at species biomass. While *D. glomerata* consistently dominated species biomass, either *P. lanceolata* or *L. corniculatus* demonstrated higher individual biomass at all climates except one. This in part is due to high survival rates of *D. glomerata*, contrasting with fewer surviving individuals of *P. lanceolata* and *L. corniculatus*. Thus, low survivorship led to higher individual competitiveness, and survival is not solely determinant of biomass dominance.

Species generally maintained their biomass hierarchies across communities while having different individual biomass hierarchies. This relationship reveals that survivorship is differentially affecting the study species within the same communities. While warming is known to influence the stability of biomass production because species respond asynchronously (Ma, Liu, et al., 2017), our findings do not support this. Instead, we find that species contributions to community productivity was relatively stable across climates. Survival was closely associated with reference soil in our study – this trend may therefore be an experimental artifact. The reference soil treatment led to higher community biomass, root biomass, and survival compared to local soils. High root biomass can indicate stronger belowground competition, with increases in belowground biomass typically being symmetric for neighboring individuals (Broadbent et al., 2018; Cahill & Casper, 2000), but this effect was not quantified here. With limited resources available in each pot, intensive root competition may have resulted in decreased species abundance, however, overall higher productivity (Rajaniemi et al., 2003; Tilman, 1990), explaining how species with lower survivorship were able to increase their individual biomass to maintain overall community hierarchies. This demonstrates how competition for space strongly affects community productivity (Schmid et al., 2021), while also agreeing with past studies finding that net primary production can be maintained even with shifts in community composition (Liu et al., 2018). Nevertheless, this observation underscores the role of survival and individual species dynamics in contributing to overall community biomass.

Individual biomass experienced the strongest positive relationship with species biomass in cases where survival was low. This reveals a survival‐influenced trade‐off, where high total species biomass is achieved with the loss of individual biomass. Thus, an individual‐rich community leads to higher net productivity in plant model communities. While this trade‐off has been documented in grassland monocultures (Chalmandrier et al., 2017; Heisse et al., 2007), this is the first documentation in plant model communities. High species evenness is considered critical in maintaining community biomass (Rohr et al., 2016). In our study, community biomass was partially maintained across communities of varying species evenness due to the limited ability of species experiencing low relative survival to produce larger individuals. This demonstrates a degree of resilience, where despite the low species evenness often observed, biomass production was still maintained at high rates.

### 4.2 Soil effects

Our study design allowed us to isolate soil effects from climatic effects on community and species‐specific trait dynamics. Surprisingly, we found that despite each study location having a unique combination of soil and climate, all communities experienced similar changes in plant traits. This community‐level finding contrasts with findings at the species level, which revealed stark variation in trait responses. Interactions between climate and species were prevalent across most response variables. A lack of distinguishable patterns in the magnitude or direction of soil effects demonstrates high trait stochasticity within our plant communities, which has previously been attributed largely to environmental variation (Davison et al., 2010; Riginos et al., 2018). Random forest analysis supported this finding, with no increases in predictive power being found when examining soil effects. Nutrient availability has also been shown to influence community assembly within grassland communities (Guo et al., 2014), with community dynamics shifting away from niche‐based determination toward stochasticity and species asynchrony in the short term under high nutrient availability (Conradi et al., 2017; Zhang et al., 2016). While our study only examined these responses following one year of treatment, our findings demonstrate the short‐term effects of nutrient variation leading to high grassland community interspecific trait stochasticity.

We accept our hypothesis that soil effects would lead to high trait variation across both species and climate. No species consistently had the largest changes for any measured trait. For example, *L. corniculatus* had greater maximum height at Stubai (1,850 m) relative to other species but grew less than others at Graswang (850 m). Furthermore, soil effects resulted in unique species variation for all traits across climates. Surprisingly, species with high values for one trait that is traditionally linked to fitness did not concurrently increase in other fitness‐linked traits. For example, if a species had high survival rates at a given climate, this did not necessitate high values for traits such as leaf dry weight, leaf area, and biomass. This offers another trade‐off example between community‐level survivorship and individual fitness, with increased survivorship potentially increasing within species competition, resulting in decreases in other fitness‐linked traits.

The combination of local soil and climate yields stochastic trait responses in our study for all species. Past experiments have documented shifts in dominance hierarchies depending on interactions between nutrient and climate treatments (Alatalo et al., 2014; S. Niu & Wan, 2008). For example, Klanderud and Totland (2005) found that climate change and nutrient addition in grassland ecosystems caused changes in dominance hierarchies, community structure, and diversity. While nutrient addition alone increased the competitiveness of graminoid and forb species, the climatic treatment did not have this effect. This aligns with our results, underscoring the role of changing species interactions resulting from variation in soil nutrient availability and composition, as well as water availability on the trait responses of individuals. It is difficult to simulate natural soil conditions, with naturally occurring soil minerals that extend well beyond standard nitrogen, phosphorous, and potassium typically included in nutrient studies. Many other micro‐ and macronutrients are known to have interactions with shifts in climate change‐relevant plant traits such as transpiration or root acquisition of soil minerals (Lynch & St. Clair, 2004). For this reason, natural system experiments remain the most complete look into the future of plant grassland communities. Given the importance of soil composition on community trait dynamics, we suggest further work investigating grassland community responses to climate change incorporate natural soil systems.

### 4.3 Climatic effects

We investigated the effect of climate on plant traits within model grassland communities using reference soil across our study climates. As indicated by our PCA analysis, species were strongly correlated with the measured traits consistently across sites, whereas climate segregation followed little to no pattern, meaning climate demonstrated minimal correlation with plant traits. Therefore, climate differences in the absence of soil differences did not lead to the restructuring of plant trait hierarchies. This finding was supported by our Random Forest analysis, leading us to reject our hypothesis that climate is the primary driver of trait variability. Instead, our results illustrate consistent responses across species even in the face of high climatic variation. Since our community was selected to minimize functional overlap, a naturally occurring community with higher functional overlap may experience less distinct trait differentiation due to direct competition (Mason et al., 2011). This could affect the application of our climate results; however, the effects of soil demonstrated no such role of functional groups in trait responses, rather revealing high stochasticity. While the interactive role of soil and climate on plant traits has been well documented in past community‐based studies (He & Dijkstra, 2014; Sundert et al., 2021), questions remain about how climate variation alone affects grassland community structures. Overall community diversity and their constituent species have a determinant role in ecosystem responses to climatic changes (Hautier et al., 2015), meaning monoculture‐based climate change experiments may not accurately represent plant trait responses. With the consistent species–species trait responses observed in our experiment, a community‐based approach may better capture predictive plant trait responses under a changing climate.

## 5 CONCLUSIONS

By using a novel experimental design, our study revealed distinct trends in community structure and species trait expression within grassland plant communities. We found that species had consistent trait responses to a variety of imposed climates. While this may suggest that climate alone does not have a strong influence on within‐community trait dynamics, it highlights the importance of the interactive role between soil and climate in the internal structure of community traits. Communities generally produced biomass in consistent hierarchies at both the community and individual scale. However, species‐specific contributions to community biomass depended heavily on soil and climate. Furthermore, in treatments where species had low survivorship, species‐specific biomass contribution was maintained through the increased biomass of surviving individuals. The effect of soil echoed this: analyses revealed stochastic variation in species trait responses across climates and species. Our integrative community‐based approach contributes to predictions of grassland ecosystem‐level changes under a changing climate, by incorporating aspects such as inter‐ and intra‐specific species responses, as well as partitioning the contributions of climate and soil. Our study offers a holistic view regarding the role of species‐level trait‐based dynamics in determining overall grassland community hierarchies.

## CONFLICT OF INTEREST

The authors have no competing interests to declare.

## AUTHOR CONTRIBUTIONS

**Franklin Alongi:** Formal analysis (lead); investigation (lead); visualization (lead); writing – original draft (lead). **Jana H. Rüthers:** Conceptualization (supporting); writing – original draft (supporting); writing – review and editing (equal). **Justyna Giejsztowt:** Conceptualization (supporting); writing – review and editing (equal). **Katrina LaPaglia:** Conceptualization (supporting); writing – review and editing (supporting). **Anke Jentsch:** Conceptualization (lead); formal analysis (supporting); funding acquisition (lead); investigation (supporting); methodology (lead); project administration (lead); writing – review and editing (equal).

### OPEN RESEARCH BADGES

This article has earned an Open Data Badge for making publicly available the digitally‐shareable data necessary to reproduce the reported results. The data is available at https://doi.org/10.5061/dryad.9s4mw6mhh.

## Data Availability

All data used in this study for direct analysis and visualization are available on Dryad via https://doi.org/10.5061/dryad.9s4mw6mhh.
